# The Library of Prof. C. C. F. Gay

**Published:** 1886-08

**Authors:** 


### *THE LIBRARY OF PROF. C. C. F. GAY.*

The widow of the late Prof. C. C. F. Gay has presented to the Niagara Medical College, of this city, the doctor's complete and valuable library, as well as his surgical instruments and a very choice botanical collection.

This is a fitting tribute to the memory of the deceased professor; a reflex of the generosity so characteristic of the doctor himself. It was in this college that he performed his last labor for the advancement of the medical profession, and on this account, no doubt, Mrs. Gay was prompted to make this valuable donation to this school. The library contains most of the standard authors; a complete set of the transactions of the American Medical Association; a complete set of the Buffalo Medical and Surgical Journal; the Medical Record for several years; the American Journal of the Medical Sciences for many years; the American reprint of the London Lancet for a number of years. In addition to this, there are many files of journals, more or less complete, and many monographs; making in all several hundred volumes, and valued at several hundred dollars. The surgical instruments are quite valuable, and the botanical collection is rare. It will, doubtless, be of great value in teaching this branch. The example of Mrs. Gay, in making such a generous presentation, should be followed by the physicians of this city and vicinity. If we are not able to contribute a large library, we can add a volume from time to time. Both of the colleges of this city have now a fine nucleus for a medical library, and the profession can donate to either, as their inclination directs, thereby assisting greatly the students in our colleges. The library of Dr. Gay is now at the college, and will be ready for the use of the students during the next term. We hope, too, the faculty of the college will open it to the profession as a consulting library.

